# Low-Cost “Telesimulation” Training Improves Real Patient Pediatric Shock Outcomes in India

**DOI:** 10.3389/fped.2022.904846

**Published:** 2022-07-26

**Authors:** Ebor Jacob G. James, Siva Vyasam, Shakthi Venkatachalam, Elizabeth Sanseau, Kyle Cassidy, Geethanjali Ramachandra, Grace Rebekah, Debasis D. Adhikari, Ellen Deutsch, Akira Nishisaki, Vinay M. Nadkarni

**Affiliations:** ^1^Pediatric Critical Care, Department of Pediatrics, Christian Medical College, Vellore, India; ^2^Pediatric Simulation Training and Research Society of India, Hyderabad, India; ^3^Center for Simulation, Advanced Education and Innovation, The Children’s Hospital of Philadelphia, Philadelphia, PA, United States; ^4^Division of Emergency Medicine, Department of Pediatrics, The Children’s Hospital of Philadelphia, Philadelphia, PA, United States; ^5^Annenberg School for Communication, University of Pennsylvania, Philadelphia, PA, United States; ^6^Department of Pediatric Intensive Care, Krishna Institute of Medical Sciences, Secunderabad, India; ^7^Department of Biostatistics, Christian Medical College, Vellore, India; ^8^Department of Anesthesiology and Critical Care Medicine, The Children’s Hospital of Philadelphia, Philadelphia, PA, United States

**Keywords:** telesimulation, simulation-based education, COVID-19 educational innovations, hotkeys, septic shock

## Abstract

**Introduction:**

Pediatric shock, especially septic shock, is a significant healthcare burden in low-income countries. Early recognition and management of shock in children improves patient outcome. Simulation-based education (SBE) for shock recognition and prompt management prepares interdisciplinary pediatric emergency teams in crisis management. COVID-19 pandemic restrictions on in-person simulation led us to the development of telesimulation for shock. We hypothesized that telesimulation training would improve pediatric shock recognition, process of care, and patient outcomes in both simulated and real patient settings.

**Materials and Methods:**

We conducted a prospective quasi-experimental interrupted time series cohort study over 9 months. We conducted 40 telesimulation sessions for 76 participants in teams of 3 or 4, utilizing the video telecommunication platform (Zoom©). Trained observers recorded time-critical interventions on real patients for the pediatric emergency teams composed of residents, fellows, and nurses. Data were collected on 332 pediatric patients in shock (72% of whom were in septic shock) before, during, and after the intervention. The data included the first hour time-critical intervention checklist, patient hemodynamic status at the end of the first hour, time for the resolution of shock, and team leadership skills in the emergency room.

**Results:**

There was a significant improvement in the percent completion of tasks by the pediatric emergency team in simulated scenarios (69% in scenario 1 vs. 93% in scenario 2; *p* < 0.001). In real patients, completion of tasks as per time-critical steps reached 100% during and after intervention compared to the pre-intervention phase (87.5%), *p* < 0.05. There was a significant improvement in the first hour hemodynamic parameters of shock patients: pre (71%), during (79%), and post (87%) intervention (*p* < 0.007 pre vs. post). Shock reversal time reduced from 24 h pre-intervention to 6 h intervention and to 4.5 h post intervention (*p* < 0.002). There was also a significant improvement in leadership performance assessed by modified Concise Assessment of Leader Management (CALM) instrument during the simulated (*p* < 0.001) and real patient care in post intervention (*p* < 0.05).

**Conclusion:**

Telesimulation training is feasible and improved the process of care, time-critical interventions, leadership in both simulated and real patients and resolution of shock in real patients. To the best of our knowledge, this is one of the first studies where telesimulation has shown improvement in real patient outcomes.

## Graphical Abstract 1

Infographics of the study. 
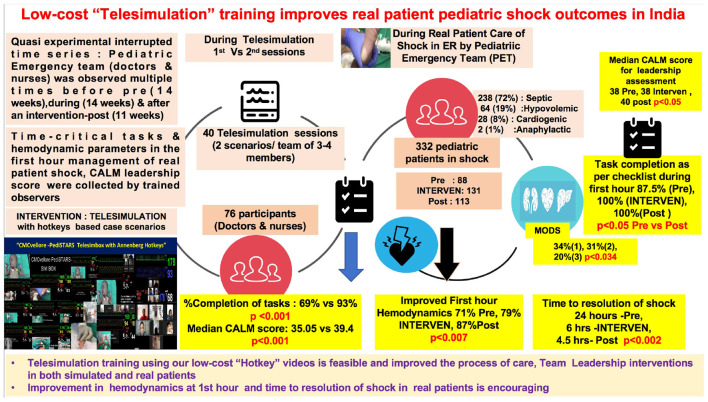

## Graphical Abstract 2

Summary of outcomes in simulated and in real patient events. 
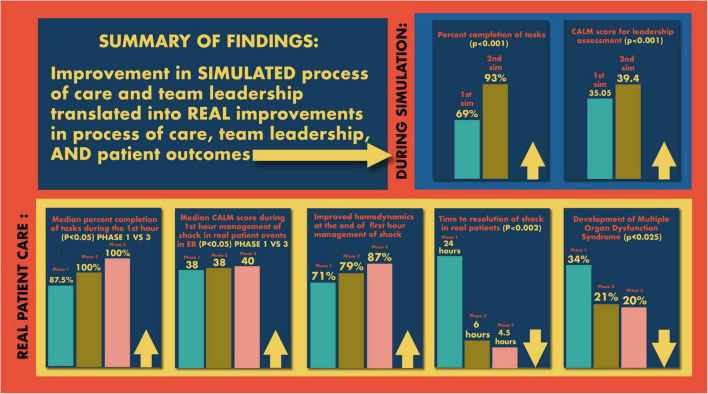

## Introduction

The leading cause of pediatric global morbidity and mortality is severe sepsis, with disparities in outcomes linked to lack of access to training and resources based on geography (1–5). The concurrent application of *in situ* simulation for training and systems analysis offers an effective strategy to improve adherence to guidelines, resulting in early diagnosis, time-critical intervention, and overall better outcomes (6, 7). Geographic and resource barriers and infection-related restrictions to *in situ* simulations can be effectively overcome by remotely facilitated simulation-based activities. Distance or remote or telesimulation is defined as “simulation performed with either the facilitator, learners, or both in an offsite location separate from other members to complete educational or assessment activities.” Facilitation and assessment can be performed either synchronously or asynchronously using video conferencing tools (8–16). The concept of telesimulation using prerecorded video was adapted from the ACEP SimBox, a free and openly accessible web-based simulation platform demonstrated to be easy to use, feasible, and recommended by healthcare team users (17–21). While the SimBox utilizes a single-track video that can be paused, rewound, or fast-forward, Annenberg hotkeys provides a random-access interface that allows a facilitator to play prerecorded videos in any order at any time, permitting a video narrative to be created on-the-fly based on input from users. This allows for simulations that can arrive at a conclusion *via* a number of different paths or with multiple interventions and outcomes.

It is known that prompt recognition and time-critical management of shock in the “golden hour” correlates with improved patient morbidity and mortality (22). Given the burden of disease of sepsis and restrictions to in-person education activities during the COVID-19 pandemic at the teaching hospital, Christian Medical College (CMC), Vellore, India, we created a low-cost telesimulation curriculum. Our aim was to train pediatric emergency teams (PET) composed of pediatric residents, fellows, and nurses in the recognition and first-hour management of shock.

The purpose of this study was to assess the impact of a telesimulation training on the PET’s first-hour management of pediatric shock. The primary objective of this study was to assess the completion of time-critical tasks (process of care) by the PET and the hemodynamic stability of real patients at the end of the first-hour management. The secondary objectives of this study were to assess time for prospectively defined shock reversal, the development of new Multiple Organ Dysfunction Syndrome (MODS), and assessment of team leadership skills in real-patient scenarios. We also assessed the completion of time-critical tasks and leadership skills during simulated scenarios. We hypothesized that our novel telesimulation training would improve the time-critical processes of care and team leadership in simulation and that this would translate to improved time-critical processes of care, team leadership, and meaningful improvement in clinical outcomes of real patients.

## Materials and Methods

The PET participated in the standard didactic series in addition to the novel telesimulation intervention described in this study. The standard didactics consist of regular lectures, case presentations, the pediatric advanced life support (PALS) course, and orientation classes on the recognition of a sick child and on the management of common pediatric emergencies, including all types of shock. The telesimulation was a novel intervention in addition to the standard curriculum. The authors wrote two scenarios, one on septic shock and another one on cardiogenic shock, with learning objectives designed to focus on the recognition, differentiation, and first-hour management of shock in pediatric patients.

### Development of the Telesimulation Platform

**FIGURE 1:**
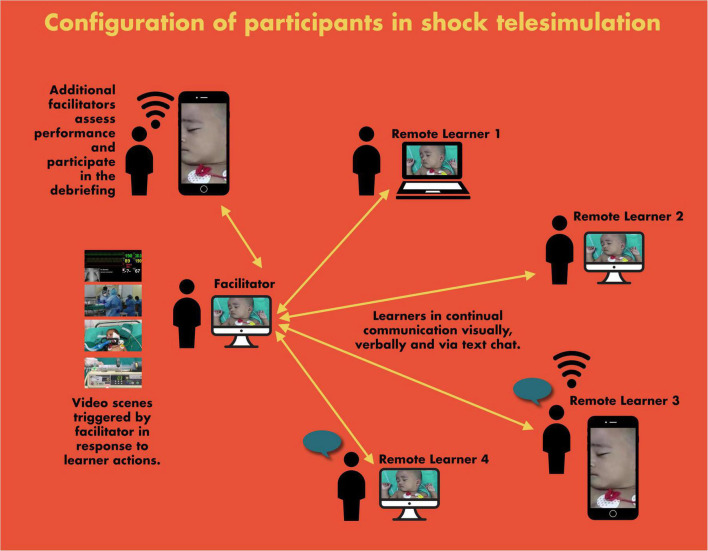
The scheme of telesimulation sessions.

**FIGURE 2:**
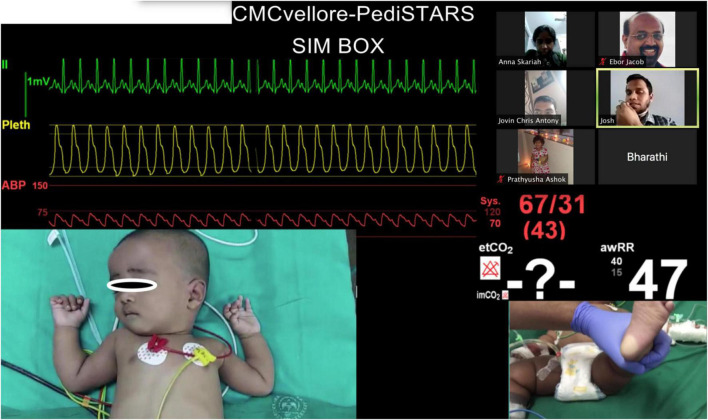
Telesimulation session showing pictures of real patient video clips with participants and facilitators meeting on the video conferencing platform Zoom©.

**FIGURE 3:**
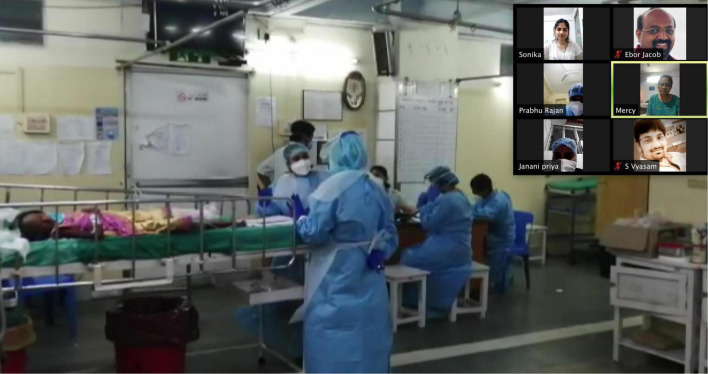
Telesimulation session showing video of a real healthcare teams in the emergency room responding to a patient with shock.

For our intervention, we created a custom module with multiple patient videos for the Annenberg hotkeys distance learning platform. Annenberg hotkeys were created in the first months of 2020 in response to the COVID-19 pandemic by technologists and filmmakers at the University of Pennsylvania’s Annenberg School for Communication in consultation with physicians from The Children’s Hospital of Philadelphia and the Perelman School of Medicine at the University of Pennsylvania. Annenberg hotkeys (23) was designed to virtualize standardized patient experiences and allow interaction between medical trainees, instructors, and prerecorded patients by allowing a facilitator to rapidly display a number of video responses based on input from learners. The Annenberg hotkeys provides a more adaptive “Choose Your Own Adventure” form of videomaking in which a facilitator can essentially edit a movie on-the-fly from a series of prerecorded clips. This gives the facilitator the ability to let learners solve problems in many ways rather than requiring them to push along a linear path. The lead author (EJ) created videos of real Indian patients using his cellphone camera, with appropriate informed consent. To protect the patient’s privacy, patient videos were off-loaded from the personal cell phone to his official computer in the department and secured. These videos were superimposed on prerecorded video clips of vital sign monitors. Annenberg hotkeys maps keys on a computer keyboard to locally stored video files. These videos will play when a key is pushed. This function works through the remote meeting platforms *via* a share-screen option. For the telesimulation activities, three facilitators and groups of three to four participants connected *via* Zoom© (Figure 1). As the learners suggested interventions, the facilitator switched between videos to display the results of those interventions. For example, when a participant saw a video of an ill patient on the screen and asked the team to perform interventions such as monitor placement, vital signs, venous access, labs, and fluid and antibiotic administration, the facilitator responded directly to each of their requests by clicking the hotkey (23) linked to the specific premade video portraying the task (Figures 2, 3).

### Inclusion and Exclusion Criteria

The PET participants included general pediatric and emergency medicine residents, fellows, and nurses working in the emergency room (ER) during the study period. Patients were eligible for the study if they were between the ages of 28 days and 15 years, as per the age cutoff for the pediatric ER in the hospital, and were presenting with clinical features of shock. Healthcare provider participants, patients, or families who did not give informed consent were excluded from the study.

### Study Design and Process

**FIGURE 4:**
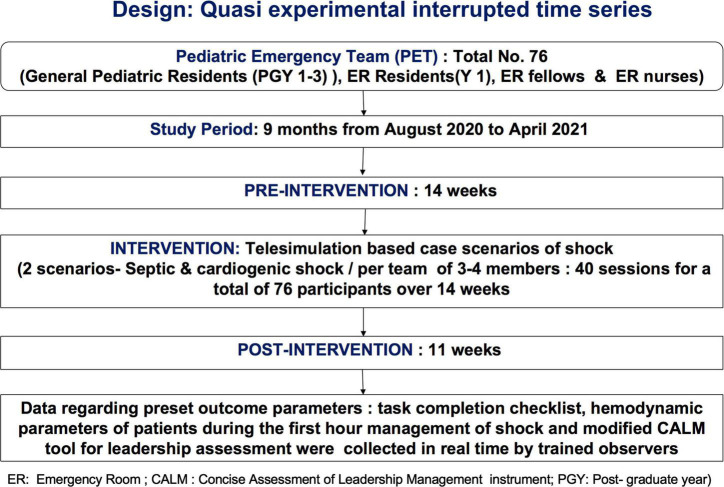
Approach and design of the telesimulation study in the emergency room.

We used a quasi-experimental interrupted time series model for our study (Figure 4). The study was approved by the Institutional Review Board of Christian Medical College (CMC), Vellore, India.

A total of 76 participants were recruited *via* convenience sampling at the local hospital, CMC, Vellore. The 9-month study period (August 2020–April 2021) was divided into three phases (Figure 4).

• Pre-intervention phase (14 weeks)—August 2020 to November 2020.
• Intervention phase (14 weeks)—November 2020 to February 2021.
• Post-intervention phase (11 weeks)—February 2021 to April 2021.

### Operational Definitions and Diagnostic Criteria

We used the standard PALS criteria to identify and classify shock. We identified patients in both compensated and hypotensive phases of shock. Patients were categorized to have compensated shock if they met the following criteria and maintained systolic blood pressure more than the age-specific 5th percentile using an automated electronic non-invasive blood pressure monitor (oscillometry) with age-appropriate cuffs.

(1) Heart rate higher than the upper limit of the age-specific normal (Supplementary Table 1), measured by an electronic monitor with adhesive electrodes.
(2) Capillary refill time >2 s, tested by applying moderate pressure to the palm, sole or finger for 5 s and measuring the time taken to return color in seconds.
(3) Cold peripheral extremities.

We defined hypotensive shock as a systolic blood pressure less than the age-specific 5th percentile. We also defined altered sensorium as a Glasgow Coma Scale (GCS) <13. Shock patients were categorized into hypovolemic, distributive, cardiogenic, and obstructive types by the PET, which was later confirmed by the investigators based on the initial clinical history, signs, and symptoms by reviewing the medical records.

Hemodynamic stabilization of the patient at the end of the first-hour management was prospectively defined if all of the following were achieved: (1) a decrease in heart rate by 5%, (2) age-specific normalization of systolic blood pressure, (3) normalization of the capillary refill time, and (4) normalization or improvement of GCS by >2 compared to baseline.

We defined multi-organ dysfunction in accordance with the international pediatric sepsis consensus conference in 2005 (24).

To assess the participant’s performance in the study, we devised a critical intervention checklist consisting of the tasks that were required to be completed by the medical team, including 8 time-critical steps (refer to Supplementary Table 4). We analyzed leadership skills and team performance using the modified CALM tool with a minimum score of 13 (worst) and a maximum score of 53 (best) (25) (Supplementary Figure 1).

### Description of Telesimulation Scenarios and Simulation Data Collection

During the intervention phase, 40 telesimulation sessions were conducted. Sessions were conducted *via* the secured video telecommunication platform Zoom© (Zoom Video Communications, San Jose, CA, United States), with all participants and facilitators remotely connecting (no two people were together in the same room for the training). Three trained facilitators (two physicians and one nurse) conducted telesimulation sessions. The lead investigator (EJ) trained the other two facilitators in a standardized way to facilitate the telesimulation. Facilitation rehearsal was conducted with meticulous planning to ensure psychological safety and to maximize learners’ interaction during debriefing (26, 27). One investigator (SV) completed observer checklists during the simulation in real time, with secondary confirmation by review of recorded sessions by the second investigator (EJ). Both of the investigators used the same checklists for the time-critical steps in the management of shock and modified CALM leadership tool.

All 76 participants participated in the telesimulation sessions two times. Each shock scenario session lasted an hour, comprised of a 10-min pre-brief, 20-min scenario, and 30-min debrief. To encourage active participation, each learner was given a role which they normally perform in real patient care. The chat box was used by other learners when one of them was talking on the Zoom© to avoid noise from too many voices. The debrief was designed to promote active reflection by the learners by encouraging plus delta by the participants followed by advocacy inquiry by the facilitators to close the gaps in performance (26). Any deficit in skills was addressed by sharing training videos on Zoom©, such as intraosseous line insertion and push–pull technique for fluid resuscitation. At the end of the session, learning points were summarized and each participant was asked to share thoughts on changes they would incorporate while caring for real patients.

As telesimulation is new to the participants, the lead investigator (EJ) conducted a 30-min virtual orientation in large groups to the participants to familiarize with the Zoom© platform and the telesimulation methodology just before the intervention phase. Simulation training consisted of small group sessions of 3–4 participants, which included pediatric residents (year 1–3), ER residents (year 1), ER fellow, and nurses working exclusively in the resuscitation bay of the ER. During the intervention period, every ER resident, general pediatric resident, ER fellows, and all nurses in the pediatric resuscitation bay participated in two telesimulation sessions on pediatric shock. Learners remained in the same teams for both sessions. The first-year pediatric and ER residents received a standard didactic educational orientation on common pediatric emergencies, including shock, during their first month of the academic year as a part of their orientation. In addition, they participated in regular lectures and bedside case discussions throughout the year.

### Data Collection for Real Patients

**FIGURE 5:**
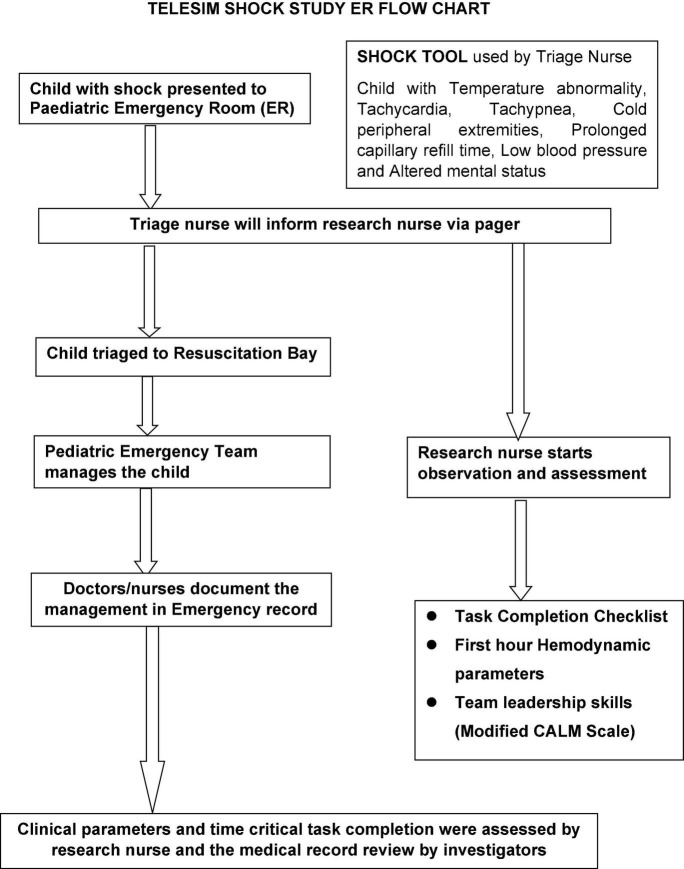
Algorithm depicting the flow of the telesimulation study in the emergency room.

Data were collected during all three phases of the study period. The pediatric intensive care unit (PICU) research nurse was alerted *via* pager by the pediatric emergency triage nurses when shock was diagnosed in a child according to the operational definitions and diagnostic criteria (Figure 5). These PICU research nurses work in shifts in the ER. They were trained in a standardized fashion to observe the performance of the team during the first-hour management and leadership skills using the modified CALM tool. Research nurses documented the team’s performance metrics using the critical intervention checklist in real time. They assessed the hemodynamic stability of children with shock during the first-hour management by collecting hemodynamic parameters in patients at regular intervals (0, 15, 30, and 60 min) during the study period. Inter-rater reliability testing was not done for these trained observers. They collected data using the same standardized checklist used for the simulated sessions (Supplementary Table 4). Later, the medical records of study patients were reviewed to note the time of shock resolution and development of MODS. The patient data, including the clinical features of shock, were recorded hourly in the ER medical records. Those children who had persistent features of shock and/or required vasoactive agents were admitted to the high dependency unit (HDU) or PICU where vital parameters were monitored and recorded hourly.

### Statistical Analysis

We analyzed critical intervention checklists completed by the research nurses for patients diagnosed with all types of shock during the 9 months study period (pre, during, and post intervention).

**FIGURE 6:**
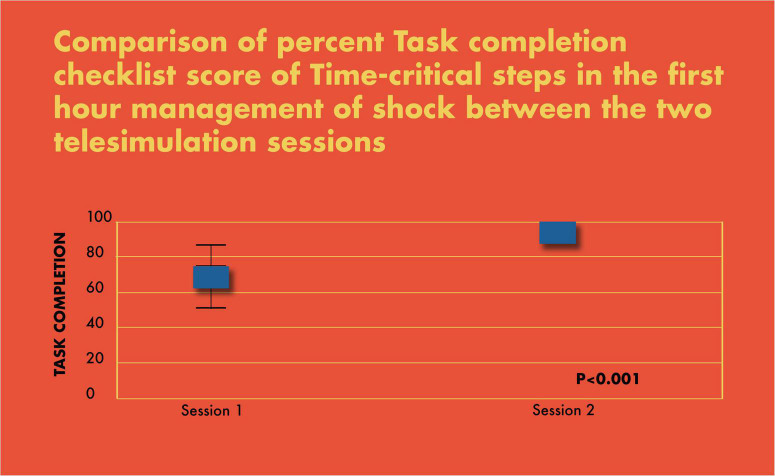
Comparison of percent task completion checklist score of time-critical steps in the first-hour management of shock between the two telesimulation sessions.

**FIGURE 7:**
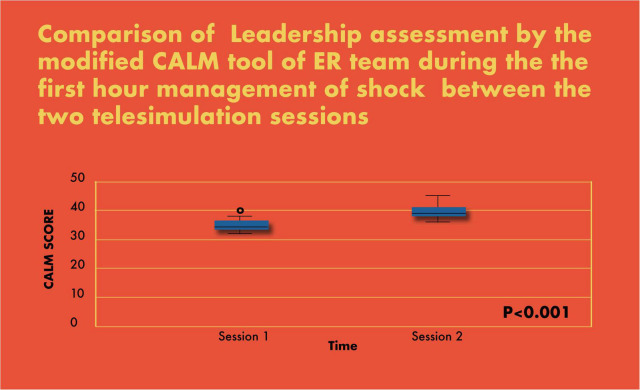
Comparison of leadership assessment by the modified CALM tool of ER team during the first-hour management of shock between the two telesimulation sessions.

In simulation assessments, the primary outcome of interest was the percent completion of tasks by the PET. The secondary outcome of interest was team leadership skills using the modified CALM tool. Both were analyzed for each team and compared between the two simulation sessions and plotted on a graph using the box and whisker plot (Figures 6, 7).

In real patient events, the primary outcome of interest was percent completion of tasks and the prospectively defined hemodynamic stability of the patient at the end of the first hour of management. Secondary outcomes included time to reversal of shock, development of MODS, and team leadership skills. Descriptive statistics for continuous variables were reported using mean ± standard deviation (SD). The Shapiro–Wilk and Kolmogorov–Simonov tests were done to see the distribution of variables. Skewed variables were reported using the median and interquartile range (IQR). Categorical variables were reported using frequency and percentage. Association was reported using chi-square or Fisher’s exact test. A comparison of means was reported using one-way ANOVA. A comparison of pattern/trend over time with respect to the baseline was reported using generalized estimating equations (GEE).

## Results

### Participant Demographics

All the 76 members of the PET which included 56 physicians comprising of all general pediatric residents (*n* = 40, year 1–3), ER residents (*n* = 10, year 1), ER fellows (*n* = 6), and all nurses (*n* = 20) participated in the study (Table 1).

**TABLE 1:** Participant demographics.

Total number of participants (*n*)	76 (100%)
Median age (in years)	28 (22–36)
Sex (*n* = 76)	
Males	25 (33%)
Females	51 (67%)
Discipline (*n* = 76)	
Emergency room resident	10 (13%)
General pediatrics resident	40 (52%)
Pediatric emergency fellow	6 (9%)
Pediatric emergency nurse	20 (26%)
Training level of residents (*n* = 40)	
Post graduate year-1	15 (37%)
Post graduate year-2	13 (32%)
Post graduate year-3	12 (31%)
PALS certified	
Physician	30 (40%)
Nurse	06 (8%)
Median years of experience (nurses, interquartile range)	2.5 (1–5)

### Patient Demographics

During the study period, 332 patients under 15 years of age with shock presented to the ER (Table 2). There were 190 out of 332 (57%) boys and 142 out of 332 (43%) girls. Of these patients with shock, 238 out of 332 (72%) were diagnosed with septic shock, 64 out of 332 (19%) with hypovolemic shock, 28 out of 332 (8%) with cardiogenic shock, and 2 out of 332 (1%) with anaphylactic shock.

**TABLE 2:** Baseline real patient characteristics.

Patient characteristics total *N* = 332	Pre-intervention phase (*n* = 88)	Intervention phase (*n* = 131)	Post-intervention phase (*n* = 113)
Sex			
Male	46 (52%)	83 (63%)	61 (54%)
Female	42 (47%)	48 (36%)	52 (46%)
Age			
<12 months	20 (23%)	41 (31%)	49 (43%)
13–60 months	22 (25%)	42 (32%)	31 (27%)
61–120 months	24 (27%)	25 (19%)	16 (14%)
>121 months	22 (25%)	23 (18%)	17 (15%)
Time seen at Triage			
8 AM to 5 PM	43 (49%)	59 (45%)	49 (43%)
5 PM to 8 AM	45 (51%)	72 (55%)	64 (57%)
Weekdays	58 (66%)	98 (75%)	84 (74%)
Weekends	30 (34%)	33 (25%)	29 (26%)
Types of shock			
Hypovolemic	16 (19%)	24 (19%)	24 (21%)
Septic	68 (78%)	93 (72%)	77 (68%)
Cardiogenic	3 (3%)	13 (10%)	12 (11%)
Obstructive	0 (0%)	0 (0%)	0 (0%)
Outcome			
Alive	79 (91%)	112 (86%)	93 (82%)
Left against medical advice	2 (2%)	12 (9%)	8 (8%)
Dead	6 (7%)	6 (5%)	11 (10%)

### Simulation Outcomes

#### Primary and Secondary Outcomes

There was a significant improvement in the completion of tasks by the PET in the simulated scenarios (69% in scenario 1 vs. 93% in scenario 2; *p* < 0.001) (Figure 6). Similarly, there was a statistically significant increase in CALM leadership scores between the first and second simulation scenarios, *p* < 0.001 (Figure 7).

### Clinical Outcomes

#### Primary Outcome

**FIGURE 8:**
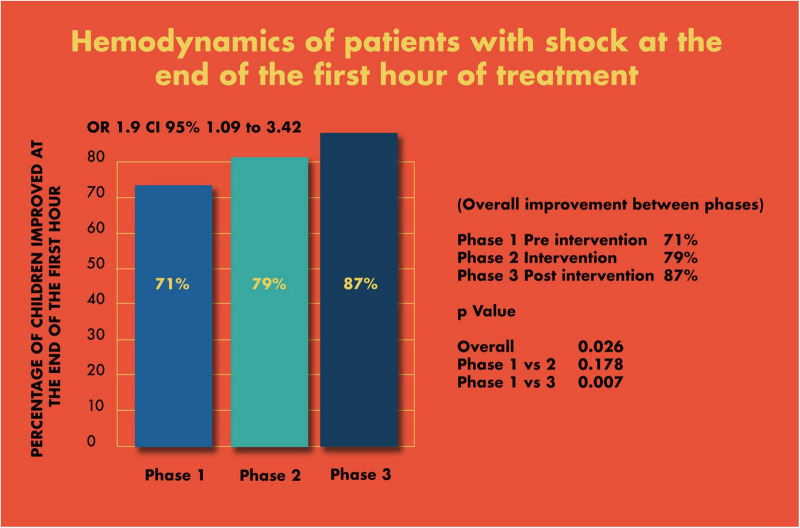
Hemodynamic stabilization at the end of the first hour.

There was a significant improvement in the first-hour hemodynamic parameters of shock patients: pre (71%), during (79%), and post (87%) intervention (*p* < 0.007 pre vs. post) (Figure 8).

#### Secondary Outcomes

**FIGURE 9:**
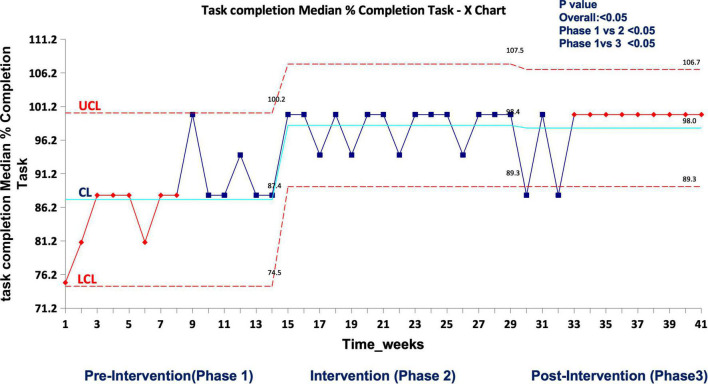
Median percent completion of task as per checklist during the first-hour management of shock in real patient events in the emergency room. UCL, upper control limit; CL, center line; LCL, lower control limit.

**FIGURE 10:**
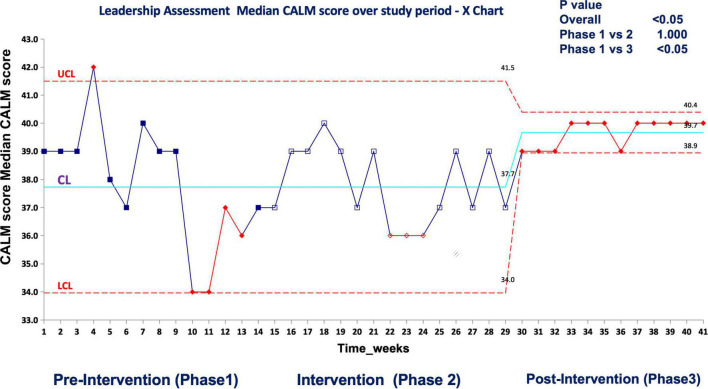
Median CALM score (leadership assessment) during the first-hour management of shock in real patient events in the emergency room UCL, upper control limit; CL, center line; LCL, lower control limit.

**FIGURE 11:**
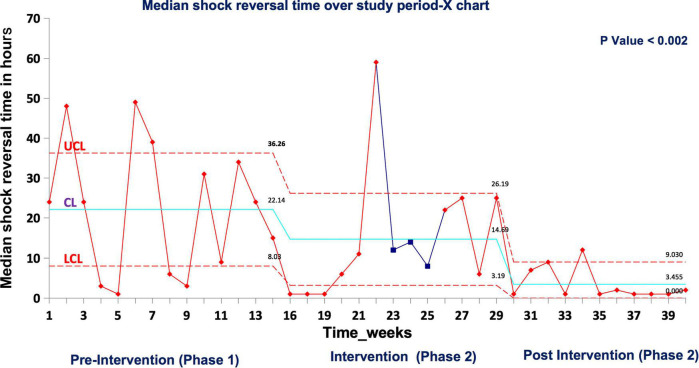
Median shock reversal time in real patient events UCL, upper control limit; CL, center line; LCL, lower control limit.

**FIGURE 12:**
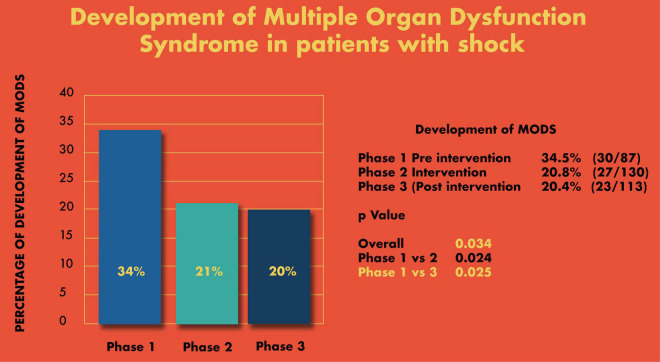
Development of Multiple Organ Dysfunction Syndrome (MODS) in patients with shock.

In real patient cases, completion of tasks as per time-critical steps reached 100% both during and post intervention compared to the pre-intervention phase (87.5%), *p* < 0.05 (Figure 9). The median-modified CALM score for leadership assessment increased after the intervention period: premedian 38, IQR (34–40); during median 38, IQR (36–39); postmedian 40, IQR (39–40); *p* < 0.05 for pre vs. post (Figure 10). Shock reversal time was reduced from 24 h (pre) to 6 h (during), and to 4.5 h (post), *p* < 0.002 for pre vs. post (Figure 11). These findings of clinical outcomes in real patients with shock are summarized in Table 3. There was a statistically significant decrease in the development of MODS in patients; 34% (pre), 21% (during), and 20% (post), *p* = 0.025 for pre vs. post (Figure 12). The survival to discharge was not different among three study phases, *p* = 0.71, pre vs. post.

**TABLE 3:** Clinical outcomes in patients with shock.

Clinical outcomes	Pre-intervention phase median (IQR)	Intervention phase median (IQR)	Post-intervention phase median (IQR)	*P*-value (pre vs. post)
Completion of time- critical task (%)	87.5 (75–87.5)	100 (87.5–100)	100 (87.5–100)	<0.05
Modified CALM Score	38 (34–40)	38 (36–39)	40 (39–40)	<0.05
Shock reversal time (hours)	24 (3–48)	6 (1–36)	4.5 (1–14)	<0.05

*CALM, concise assessment of leadership management. Shock reversal time: time of resolution of shock.*

## Discussion

Our study assessed the impact of telesimulation-based training of PET on time-critical management of the first hour of shock, team leadership in both simulated and real-world contexts, and real patient outcomes in the ER. We showed a statistically significant improvement in the completion of the critical tasks by the team in the first hour during the intervention and post-intervention phases when compared to the pre-intervention phase, in both simulated and clinical environments. Most importantly, we demonstrated remarkable improvement in the hemodynamic stability, faster shock reversal time, decreasing MODS, and improved team leadership in real patients over the course of our study period. There are many barriers to quality simulation-based medical education, including resources, time, and geography. We propose that this low-cost telesimulation approach has the potential to successfully train healthcare teams, including in low- and middle-income countries, and ultimately improve real patient outcomes.

For decades, on-site simulation-based education (SBE), where all learners and facilitators are together, has demonstrated positive outcomes in healthcare (28, 29). However, few studies have been conducted to assess the effectiveness of telesimulation in patient care. In addition, access to on-site simulation can be difficult and costly in low- and middle-income countries, especially in rural and remote areas. The use of varying telesimulation methods has grown exponentially to meet demand during the COVID-19 pandemic. Studies have proven that telesimulation is feasible to do, and learners and facilitators perceive it to be an effective learning experience (30–34). However, to the best of our knowledge, no studies have assessed the impact of telesimulation on patient outcomes (Kirkpatrick levels 3 and 4) (35). Our study not only improved time-critical management during simulation but also improved the management and outcome of patients. This is very encouraging because similar low-cost telesimulation projects targeting other clinical interventions could be widely implemented in resource limited settings to improve patient care.

The importance of the first-hour management of shock, including recognition and treatment, has been proven to decrease mortality and influence patient outcomes (36, 37). We had planned our on-site simulation training before the COVID-19 pandemic. We adapted to pandemic restrictions by seeking international collaboration to develop an alternative approach to conduct simulation. Despite the challenge, our pandemic-proof intervention leveraging telesimulation with new Annenberg hotkey technology turned out to be highly effective in improving processes of care and outcomes for children in the ER.

### Challenges

It was difficult to identify patients in shock to videotape with parental consent. Despite this, we were able to find several patients and families willing to participate. During the intervention phase, it required tremendous coordination effort to ensure that the participating team consisted of multidisciplinary learners and the same team returned to the second scenario. Training the PICU research nurses for data collection on clinical shock cases and ensuring their availability around the clock needed a high level of collaboration with the medical and nursing leadership team.

### Limitations

The findings of this study must be interpreted with consideration of certain limitations. This study was conducted over 9 months with data collected pre, during, and immediately post-simulation intervention. During the 14 weeks of the intervention period, a total of 40 telesimulations were conducted. We cannot make an inference about what dose and frequency of training is necessary and whether it is feasible to create long-lasting, sustainable results. We had to choose a pragmatic dose and frequency of training for this study. Additionally, trained research nurses recorded the clinical interventions in real time. They were trained in data collection, especially to observe the preselected time-critical steps, leadership skills (modified CALM tool), and record hemodynamic parameters of the real patients. We did not assess their inter-rater reliability, which may have an impact on the consistency of data collection. Although these research nurses were present in the resuscitation bay during all emergencies, it is possible that the presence of the research nurse triggered the PET to remember shock and improve their clinically relevant reaction times (i.e., Hawthorne effect). Therefore, it is hard to infer if the same teams would have been this quick to react in the absence of an active observer. Nevertheless, the research team’s presence in our ER is not new. The research nurses have regularly collected data on several ongoing studies for many years, and the PET are used to their presence. We do expect that there may be a chance of some improvement in the CALM score with the ongoing clinical experience of the team members.

This is a single-center study in a tertiary pediatric academic hospital in India, conducted by investigators who are experienced simulation educators. It is yet to be explored if our results would be reproducible if conducted in different resource-limited settings.

Future work will include expanding our telesimulation training to reach providers working in the ten remote mission hospitals, which are part of the CMC hospital referral network across India. We are in the process of conducting a needs assessment to create new emergency scenarios commonly seen at primary care hospitals. More work is needed to demonstrate dose, frequency, and type of education on different levels of learners in different clinical settings. Our first step is to identify local champions at the remote mission hospitals to train as telesimulation facilitators and conduct their own drills for their healthcare teams. This would be the next step to evaluate if novice and newly trained facilitators can achieve similar results with both simulation and real patient outcomes in remote settings.

## Conclusion

Our study has demonstrated that telesimulation using prerecorded video clips with innovative hotkey functions through multicenter collaboration is an innovative, low-cost solution in response to pandemic restrictions in resource-limited settings. Telesimulation training was feasible and improved time-critical interventions, leadership in both simulated and real patients, and clinical outcomes in real patients.

## Data Availability Statement

The raw data supporting the conclusions of this article will be made available by the authors, without undue reservation.

## Ethics Statement

The studies involving human participants were reviewed and approved by the Institutional Review Board, Christian Medical College Hospital, Vellore, India. Written informed consent to participate in this study was provided by the participants’ legal guardian/next of kin. Written informed consent was obtained from the minor(s)’ legal guardian/next of kin for the publication of any potentially identifiable images or data included in this article.

## Author Contributions

EJ, SVy, ES, GRa, and VN contributed to curriculum development, faculty rehearsal, and ongoing support for training. KC contributed to creating hotkeys. EJ created and edited real patient videos to make customized telesimulation scenarios. EJ and SVy conducted all telesimulation sessions. GRe supported with statistical analysis. DDA supported with the logistics of the Pediatric Emergency Team and in organizing simulation sessions. SVe contributed to the first draft of the manuscript. ED and KC helped with graphics and figures. All authors contributed and provided critical revisions, and approved the current version.

## Conflict of Interest

The authors declare that the research was conducted in the absence of any commercial or financial relationships that could be construed as a potential conflict of interest.

## Publisher’s Note

All claims expressed in this article are solely those of the authors and do not necessarily represent those of their affiliated organizations, or those of the publisher, the editors and the reviewers. Any product that may be evaluated in this article, or claim that may be made by its manufacturer, is not guaranteed or endorsed by the publisher.
