# Supplementary material for: Low-Cost “Telesimulation” Training Improves Real Patient Pediatric Shock Outcomes in India
Source: Front Pediatr. 2022 Jul 26;10:904846. doi: 10.3389/fped.2022.904846 (PMC9364444; doi:10.3389/fped.2022.904846)
Supplement: Supplementary file 1 [file Data_Sheet_1.PDF]

Table S1: Normal heart rate for age (PediatrCrit Care Med 2007; 8:138 –144)

Age Group	Heart Rate (beats per minute)
Term newborn	120–180
Up to 1 yr	120–180
Up to 2 yrs	120–160
Up to 7 yrs	100–140
Up to 15 yrs	90–140

Table S2: Calculated 5th percentile systolic blood pressure (mm Hg) according to height percentiles among boys (M) and girls (F) 1–18 years old (PediatrCrit Care Med 2007; 8:138 –144)

Fifth Percentile Systolic Blood Pressure, Percentile for Height

	5th		25th		50th		75th		95th	
**Age (in years)**	**M**	**F**	**M**	**F**	**M**	**F**	**M**	**F**	**M**	**F**
1	62	66	65	68	67	68	70	71	72	73
2	67	68	70	70	70	71	72	71	74	73
3	68	68	71	71	73	71	76	74	77	76
4	70	71	73	73	75	74	78	74	79	76
5	72	71	76	74	78	76	78	77	80	79
6	73	74	76	76	78	77	81	79	83	81
7	74	76	77	78	79	79	81	79	83	82
8	77	78	80	78	82	81	82	82	84	84
9	77	78	80	81	82	83	85	84	87	86
10	79	80	83	83	85	85	85	86	89	88
11	81	82	85	85	87	85	87	88	89	90
12	83	85	86	87	89	87	91	90	93	92
13	87	87	88	89	90	90	92	92	94	92
14	88	89	91	89	94	92	96	93	98	95
15	92	90	95	92	95	93	97	93	99	95
16	93	91	96	93	98	93	101	96	103	98
17	97	91	98	93	100	93	102	96	104	98

Table S3: PEDIATRIC GLASSGOW COMA SCALE

PEDIATRIC GLASGOW COMA SCALE (PGCS)				
	**> 1 Year**		**< 1 Year**	**Score**
**EYE OPENING**	Spontaneously		Spontaneously	4
	To verbal command		To shout	3
	To pain		To pain	2
	No response		No response	1
**MOTOR RESPONSE**	Obeys		Spontaneous	6
	Localizes pain		Localizes pain	5
	Flexion-withdrawal		Flexion-withdrawal	4
	Flexion-abnormal (decorticate rigidity)		Flexion-abnormal (decorticate rigidity)	3
	Extension (decerebrate rigidity)		Extension (decerebrate rigidity)	2
	No response		No response	1
	**> 5 Years**	**2-5 Years**	**0-23 months**	
**VERBAL RESPONSE**	Oriented	Appropriate words/phrases	Smiles/coos appropriately	5
	Disoriented/confused	Inappropriate words	Cries and is consolable	4
	Inappropriate words	Persistent cries and screams	Persistent inappropriate crying and/or screaming	3
	Incomprehensible sounds	Grunts	Grunts, agitated, and restless	2
	No response	No response	No response	1
			**TOTAL PEDIATRIC GLASGOW COMA SCORE (3-15):**	

Table S4 a: Checklist of steps in the management of Shock

Sl.No	INTERVENTIONS	Task Initiated Y/N	TIME
1	Time seen at triage		
2	Time from triage to resuscitation bay		
3	Time seen by Resuscitation Room (RR) nurse		
4(T0)	Time to complete initial assessment (S/B doctor)		
5	Identifying shock as per ACCM-PALS guidelines<br>Time to verbalize		
6	Initiate oxygen		
7	Time to secure Ist Vascular access (IV/ IO) after<br>recognizing Shock Attempts: ______		
8	Time to administer first fluid bolus<br>(Volume: 20ml/kg or 10ml/kg or 5ml/kg<br>Duration:_________; Technique: Pull & Push or IV Flow		
9	Reassessment Performed YES /NO		
10	Time to administer second fluid bolus?<br>Volume: 20ml/kg or 10ml/kg or 5ml/kg<br>Duration:______________; Technique:		
11	Reassessment Performed YES /NO		
12	Time to administer third fluid bolus ?<br>Volume: 20ml/kg or 10ml/kg or 5ml/kg<br>Duration:______________; Technique:		
13	Reassessment Performed YES /NO		
14	Obtain blood culture prior to antibiotics YES/NO		
15	Time (start time) to give first dose of antibiotics		
16	Obtain Blood Glucose YES/NO		
17	Differentiate type of shock YES/NO<br>Hypovolemic/ Distributive/Cardiogenic/ Obstructive		
18	Initiation of Vasoactive agent YES /NO<br>Adrenaline / Noradrenaline / Dopamine / Dobutamine		
19	Obstruction relieved YES/ NO		

Table S4 a: TELESIM Septic Shock time-critical steps Checklist

Study No. **Hosp. no:** **Date:**

SERIAL NO	INTERVENTION Done	YES (+1)	NO (0)
1	Initial Assessment done		
2	Identifying as Shock / Sepsis		
3	Initiate oxygen(Hi-flow/low flow)		
4	Initiate Vascular Access		
5	Blood Culture /Labs		
6	1st fluid bolus (Pull and push)		
7	Reassessment done		
8	IV antibiotics with dose		
	Total score	/ 8	

Table S4 b: TELESIM Hypovolemic Shock time-critical steps checklist

Study No. **Hosp. no:** **Date:**

SERIAL NO	INTERVENTION Done	YES (+1)	NO (0)
1	Initial Assessment done		
2	Identifying shock /differentiating as Hypovolemic shock		
3	Initiate oxygen(Hi-flow/low flow)		
4	Initiate Vascular Access		
5	Labs		
6	1st fluid bolus (Pull and push)		
7	Reassessment done		
8	2nd Fluid Bolus		
	Total score	/ 8	

Table S4 c: TELESIM Cardiogenic Shock time-critical steps checklist

Study No. Hosp. no: Date:

SERIAL NO	INTERVENTION Done	YES (+1)	NO (0)
1	Initial Assessment done		
2	Identifying shock / differentiating as cardiogenic shock		
3	Initiate oxygen(Hi-flow/low flow)		
4	Initiate Vascular Access		
5	Labs		
6	1st fluid bolus		
7	Reassessment done		
8	Early inotropes +/- Respiratory support		
	Total score	/ 8	

Table S4 d : TELESIM Obstructive Shock time-critical steps checklist

Study No. Hosp. no: Date:

SERIAL NO	INTERVENTION Done	YES (+1)	NO (0)
1	Initial Assessment done		
2	Identifying as Septic shock		
3	Initiate oxygen(Hi-flow/low flow)		
4	Initiate Vascular Access		
5	Labs		
6	1st fluid bolus (Pull and push)		
7	Reassessment done		
8	Relieving Obstruction		
	Total score	/ 8	

Table S5: **Hemodynamic parameters**

**HEMODYNAMIC PARAMETERS**	**0 Min.**	**15 Min.**	**30 Min.**	**45 Min.**	**IMPROVED (YES/NO)**	**3rd hour**	**6th hour**	**Shock Reversal Time***
**HR (bpm)**								
**Rhythm**								
**SBP(mmHg)**								
**DBP(mmHg)**								
**MAP(mmHg)**								
**CRT**								
**GCS**								
**Respiratory rate**								
**Gallop**								
**Liver size**								

***Shock reversal time : Time of resolution of shock (Normal perfusion/ MAP/ HR for age without usage of vasopressors)**

CALM SCALE

1)LEADERSHIP

A. Role

1. Announced role as a leader ☐ no (0) ☐ yes (1)

2. Clear role as a leader throughout the case ☐ rarely (1) ☐ sometimes (2) ☐ mostly (3) ☐ always (4)

2)COMMUNICATION

A. Voice is appropriately loud and clear ☐ rarely (1) ☐ sometimes (2) ☐ mostly (3) ☐ always (4)

B. Address people explicitly ☐ rarely (1) ☐ sometimes (2) ☐ mostly (3) ☐ always (4)

C. Reinforces closed loop communication ☐ rarely (1) ☐ sometimes (2) ☐ mostly (3) ☐ always (4)

3)TEAM MANAGEMENT

A. Assigns or acknowledges roles ☐ rarely (1) ☐ sometimes (2) ☐ mostly (3) ☐ always (4)

B. Assigns tasks/Directs team effectively ☐ rarely (1) ☐ sometimes (2) ☐ mostly (3) ☐ always (4)

C. Balances workload of team ☐ rarely (1) ☐ sometimes (2) ☐ mostly (3) ☐ always (4)

D. Engages team members in decision making ☐ rarely (1) ☐ sometimes (2) ☐ mostly (3) ☐ always (4)

E. Summarizes case status periodically ☐ rarely (1) ☐ sometimes (2) ☐ mostly (3) ☐ always (4)

4)MEDICAL MANAGEMENT

A. Prioritizes task order ☐ rarely (1) ☐ sometimes (2) ☐ mostly (3) ☐ always (4)

B. Periodically reassess patient ☐ rarely (1) ☐ sometimes (2) ☐ mostly (3) ☐ always (4)

C. States next step in patient care ☐ rarely (1) ☐ sometimes (2) ☐ mostly (3) ☐ always (4)

D. Aware of limitations and seeks help as needed ☐ rarely (1) ☐ sometimes (2) ☐ mostly (3) ☐ always (4)

Maximum score : 53, Minimum Score: 13

Supplement Figure (SF) 1. Assessment of Leadership skills and teamwork using the Modified CALM Tool [26]
